# Efficacy and safety of photodynamic therapy for non–muscle-invasive bladder cancer: a systematic review and meta-analysis

**DOI:** 10.3389/fonc.2023.1255632

**Published:** 2023-10-04

**Authors:** Haitao Li, Gongwei Long, Jun Tian

**Affiliations:** Department of Urology, National Cancer Center/National Clinical Research Center for Cancer/Cancer Hospital & Shenzhen Hospital, Chinese Academy of Medical Sciences and Peking Union Medical College, Shenzhen, China

**Keywords:** photodynamic therapy, non-muscle-invasive bladder cancer, BCG therapy, bladder preserving, systematic review, meta-analysis

## Abstract

**Background:**

Photodynamic therapy (PDT) is a promising treatment for non-muscle-invasive bladder cancer (NMIBC), we conducted this systematic review to comprehensively assess its efficacy and safety.

**Methods:**

A comprehensive literature research was conducted using PubMed, Web of Science, and Scopus, and studies reporting the safety and efficacy of PDT in NMIBC were included. Complete response (CR) rates, recurrence-free survival (RFS) at different time points, and complication incidences were extracted and synthesized. Pooled results were presented as rates with a 95% confidence interval (95% CI).

**Results:**

Overall, 28 single arm studies were included in the meta-analysis. For unresectable NMIBC, therapeutic PDT achieved CR in 68% (95% CI: 59%-77%) of patients. Among these CR cases, 71% (95% CI: 56%-85%) and 38% (95% CI: 12%-64%) have a RFS longer than 12 and 24 months, respectively. For Tis patients, the CR rate was 68% (95% CI: 56%-80%), and 84% (95% CI: 48%-100%) and 13% (95% CI: 1%-32%) have a RFS longer than 12 and 24 months. For patients with resectable tumors, post-resection adjuvant PDT could provide a 12 and 24 months RFS in 81% (95% CI:76%-87%) and 56% (95% CI:41%-71%) of them. Especially, for NMIBC patients who failed BCG therapy, adjuvant PDT could still achieve a 1-year and 2-year RFS in 68% (95% CI:51%-86%) and 56% (95% CI:32%-81%) patients. The complications were mostly mild and transient, including lower urinary tract symptoms and photosensitivity.

**Conclusion:**

Both therapeutic and adjuvant PDT present satisfying safety and efficacy for NMIBC, including these cases that are resistant to the standard of care. As a promising option for NMIBC, PDT deserves further exploration by future high-quality research.

**Systematic review registration:**

https://inplasy.com/inplasy-2022-11-0043/, INPLASY2022110043.

## Introduction

Bladder cancer (BCa) is one of the most common urological malignancies. In 2022, 81,180 new BCa cases and 17,100 BCa deaths are projected to occur in the United States, according to data from the American Cancer Society (1). Approximately 75% of newly diagnosed BCa is non-muscle-invasive bladder cancer (NMIBC), which is defined as tumors confined to the bladder mucosa or submucosa (2).

The transurethral resection of bladder tumor (TURBT) is the standard strategy for NMIBC, followed by intravesical instillation of chemotherapy agents or bacillus Calmette-Guérin (BCG) to prevent the recurrence (3, 4). Nevertheless, about half of NMIBC patients might experience recurrence during follow-up after standard treatment (5, 6). When NMIBC repeatedly recurs, even progress in some cases, radical cystectomy (RC) would be inevitable, which could severely impair the quality of life. Furtherly reducing the recurrence rate has been a continuing and essential topic in the management of NMIBC.

Photodynamic therapy (PDT) is based on the local or systemic application of a photosensitive compound - the photosensitizer, which is intensely accumulated in pathological tissues (7). The photosensitizer could be activated by light of appropriate wavelength to selectively destruct tumor cells. Good therapeutic results and the possibility of the parallel application of PDT with other therapeutic protocols allow it to be commonly used in several cancers including esophageal cancer, lung cancer, and BCa (8).

Since the first report of PDT in NMIBC by Benson et al. in 1983 (9), several clinical studies have been conducted to explore the application of PDT in BCa. Furthermore, for NMIBC patients who experienced several recurrences after intravesical chemotherapy and BCG, PDT could still be an alternative option.

As a promising therapeutic option for NMIBC, the application of PDT is still arbitrary in clinical practice. To the best of our knowledge, no systematic review or meta-analysis is available to provide an analysis of the clinical utility of PDT in NMIBC. Therefore, we conducted this systematic review to comprehensively summarize the relevant clinical studies, and assess the efficacy and safety of PDT in the treatment of NMIBC.

## Methods

This systematic review and meta-analysis were conducted according to the Preferred Reporting Items for Systematic Reviews and Meta-Analyses (PRISMA) statement (10, 11). The protocol of the systematic review has been prospectively registered on Inplasy (Register ID: INPLASY2022110043).

### Literature search and screening

A comprehensive literature search was conducted for studies published from the inception of databases to 13 Nov 2022, in PubMed, Web of Science, and Scopus to identify relevant studies.

Separate searches were performed using keywords including “photodynamic”, “photodynamic therapy”, “bladder cancer” and “bladder tumor”. The inclusion criteria are as follows: (1) pathologically confirmed NMIBC; (2) included > 5 patients who received PDT; (3) clinical studies including randomized-controlled trials, case-control studies, and single-arm reports; (4) efficacy and/or safety results were reported; (5) follow-up duration no less than 6 months, if efficacy was reported; (6) report was written in English. Articles would be excluded if: (1) review, editorials, meeting abstract, and other literature without original data; (2) follow-up data was unavailable; (3) duplicated report.

Titles and abstracts of articles identified by the keyword search were screened against the study selection criteria. Potentially relevant articles were evaluated with the full text. An additional manual search of references from identified studies was performed. Two independent reviewers screened all studies according to inclusion and exclusion criteria, and all disagreements were resolved by discussion with a third author.

### Data extraction and quality assessment

Two reviewers independently extracted data from every study and evaluated methodological quality. The following information was extracted from each study if available: study design; region of study; the number of cases; inclusion criteria; T stage; pathological grade; photosensitizer; administration of photosensitizer; total laser energy; simultaneously adjuvant therapy; complications and efficacy.

In reports of therapeutic PDT, in which the PDT was applied to eliminate unresectable lesions, the complete response (CR) rates at 3 months after PDT would be extracted as efficacy indicators. The long-term recurrence-free survival (RFS) status of CR cases would also be extracted if available.

For adjuvant PDT, in which the visible lesions were resected prior to PDT and PDT was conducted for prophylaxis of recurrence, the long-term RFS status would be collected as efficacy indicators.

For reports that contain the data of the same cohort, the most informative-abundant and updated version will be included and analyzed.

The quality of reports was assessed by two reviewers independently using Joanna Briggs Institute (JBI) critical appraisal checklist for case series (12).

### Data analysis

The normality of response rates was tested by the Shapiro-Wilk normality test. Freeman-Tukey Double arcsine transformation would be performed if the response rates did not fit the normal distribution. A fixed-effects model was used to calculate the pooled estimates if no significant heterogeneity was identified (*I* ^2^<50%). Otherwise, a random-effects model was used. The pooled results were presented as CR rate with a 95% confidence interval (95% CI). Additionally, a sensitivity analysis was also performed by changing the effect model. All analyses were conducted by the “meta” package on R software (version 4.1.1) (13).

## Results

### Article search and selection

**Figure 1:**
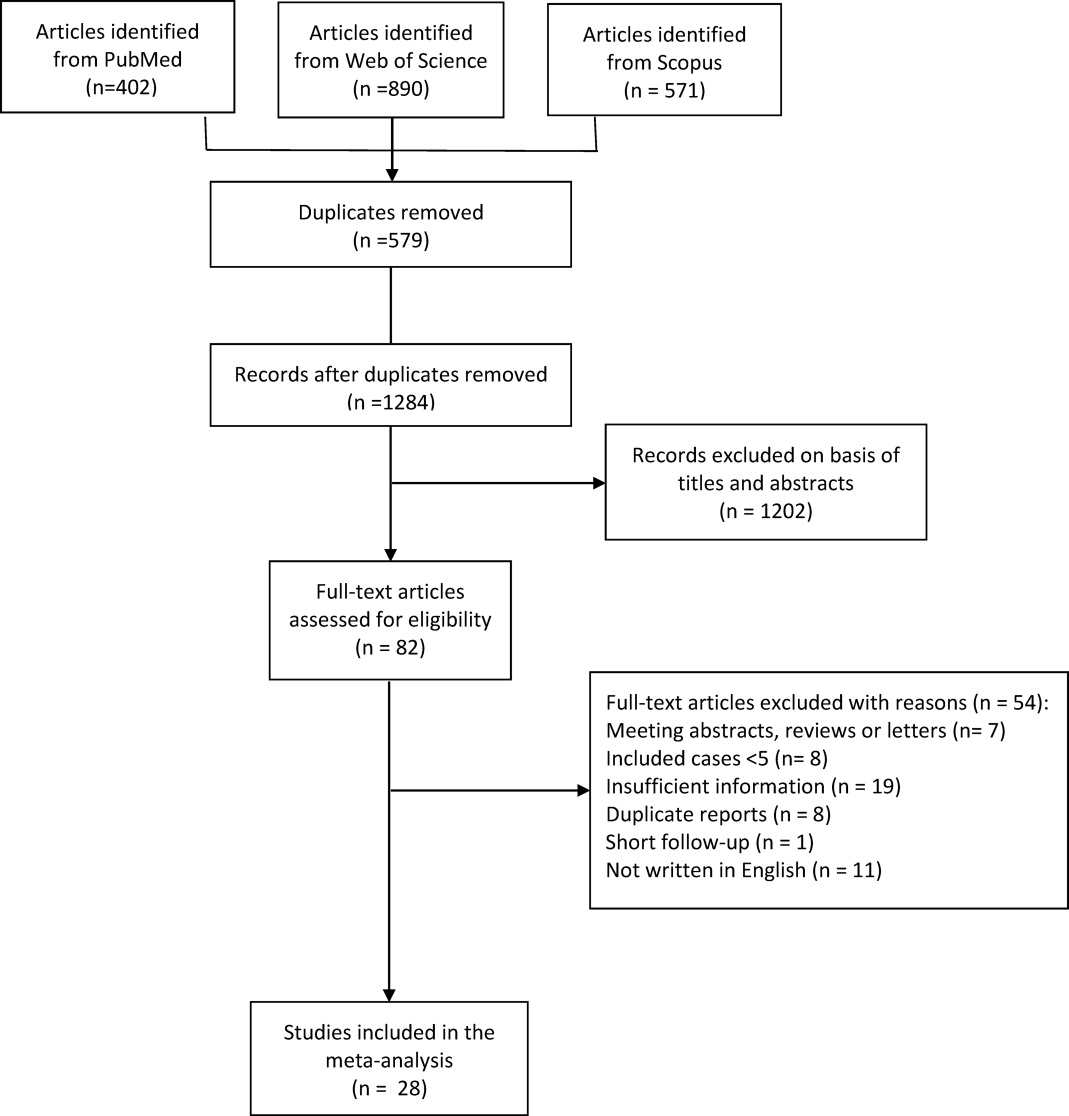
The flow chart of the literature screening.

After literature searching, 402, 890, and 571 records were identified from PubMed, Web of Science, and Scopus, respectively (**Figure 1**). After de-duplication, 579 duplicates were removed, and 1284 records were eligible for further screening based on titles and abstracts.

After a preliminary screening of titles and abstracts, 82 articles were included for full-text assessment. After full-text evaluation and data extraction, 28 articles were finally included in the meta-analysis (14–41).

**Figure 2:**
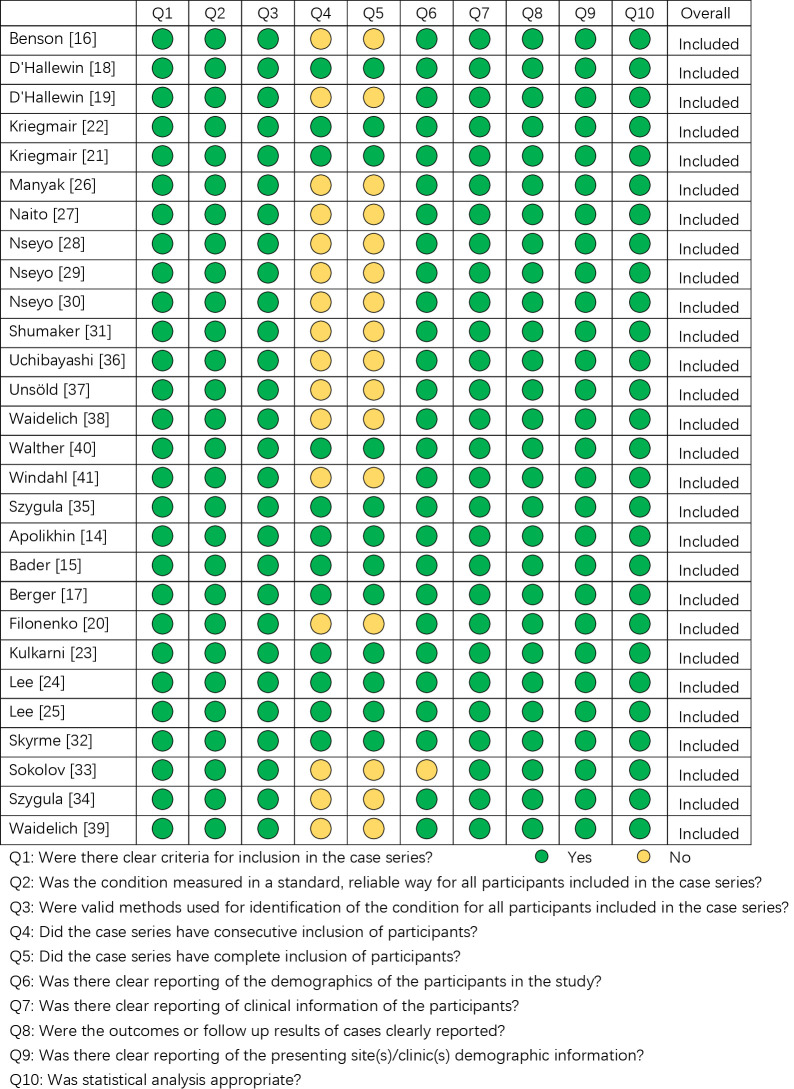
The quality assessment of the included studies according to Joanna Briggs Institute (JBI) Critical Appraisal Checklist for Case Series.

The quality assessment was conducted using the JBI critical appraisal checklist for case series. As shown in **Figure 2**, all included studies have acceptable quality as case series studies.

### Characteristics of included literature

As seen in **Table 1**, 17 articles reported the relevant outcomes of therapeutic PDT. All of these studies were conducted between 1985 – 2005. Photofrin and 5-aminolevulinic acid (5-ALA) were both widely used. Photofrin was mainly given intravenously, while 5-ALA was both intravenously and intravesical administrated. All of these reports were single-arm studies with limited sample sizes.

**Table 1:** Characteristics of reports of therapeutic PDT.

Author	Year	Region	Photosensitizer	Administration	No. of cases	Included patients	post-BCG (%)	Tis (%)	Light energy (J/cm^2^)
Benson (16)	1986	USA	Photofrin	Intravenous	10	Multifocal Tis	0	10/10	24-45
D’Hallewin (18)	1995	Belgium	Photofrin	Intravenous	15	Multifocal Tis	3/15	15/15	75-100
D’Hallewin (19)	1997	Belgium	5-ALA	Intravesical	6	Recurrent Tis	6/6	6/6	75
Kriegmair (22)	1995	Germany	Photofrin	Intravenous	21	Recurrent, multifocal NMIBC	NA	5/21	15-30
Kriegmair (21)	1996	Germany	5-ALA	Intravesical	10	Recurrent NMIBC	10/10	4/10	15-60
Manyak (26)	2003	USA	Photofrin	Intravenous	34	Recurrent NMIBC	30/34	29/34	20-25
Naito (27)	1991	Japan	Photofrin	Intravenous	35	Recurrent NMIBC	NA	29/35	10-30
Nseyo (28)	2003	USA	Photofrin	Intravenous	5	Recurrent NMIBC	NA	2/5	10-60
Nseyo (29)	1998	USA	Photofrin	Intravenous	39	Recurrent NMIBC	Most	20/39	10-60
Nseyo (30)	1998	USA	Photofrin	Intravenous	36	Recurrent Tis	36/36	36/36	15
Shumaker (31)	1986	UK	Photofrin	Intravenous	14	Recurrent Tis	NA	14/14	15-25
Uchibayashi (36)	1995	Japan	Photofrin	Intravenous	34	Recurrent Tis	NA	34/34	NA
Unsöld (37)	1990	Germany	Photofrin	Intravenous	20	Recurrent Tis	NA	20/20	15-70
Waidelich (38)	2003	Germany	5-ALA	Intravesical	11	Recurrent, multifocal NMIBC	NA	9/11	100
Walther (40)	1997	USA	Photofrin	Intravenous	20	Recurrent NMIBC	5/20	6/20	16.5-25.6
Windahl (41)	1993	Sweden	Photofrin	Intravenous	10	Refractory NMIBC	1/10	8/10	15-53
Szygula (35)	2001	Poland	5-ALA	intravesical	12	NMIBC	NA	5/12	10

Tis, Tumor in situ; 5-ALA, 5-aminolevulinic acid; NMIBC, Non-muscle-invasive bladder cancer.

The included patients’ characteristics of included studies were summarized in **Table 1**. Generally, therapeutic PDT was applied to recurrent or multifocal, unresectable NMIBC. 1^st^ generation photosensitizers (mixtures deviated from hematoporphyrin, such as photofrin) were intravenously administrated and 2^nd^ generation photosensitizers (compounds such as 5-ALA) were intravesically administrated in these studies. Especially, about 3/4 of included patients have concurrent Tis, and included patients were recurrent NMIBC in 14 of 17 included studies. Therapeutic PDT was experimentally applied to these patients to eliminate visible lesions, as an alternative to RC.

**Table 2** summarized the characteristics of adjuvant PDT reports. 12 articles published between 1998 and 2022 reported the relevant outcomes of adjuvant PDT. Different generations of photosensitizers were applied including several novel agents such as hexaminolevulinate (HAL) and radachlorin. Intravenous and intravesical administration were both widely used, and oral agents were also explored in a trial. As for included patients, adjuvant PDT was usually applied in high-risk NMIBC, especially in BCG-unresponsible patients, to prevent recurrences. 72.4% (229/316) of included patients were classified as high-risk. In 8 studies, the amount of post-BCG patients were reported, and the proportion is 71.7% (109/152). Specially, in Filonenko’s (20) and Lee’s (24) study, TURBT and PDT were simultaneously conducted. Within 2 to 3 hours after intravenous photosensitizer infusion (24) or 1.5 to 2 hours after intravesical instillation (20), TURBT was performed, followed by PDT.

**Table 2:** Characteristics of reports of adjuvant PDT.

Author	Year	Region	Photosensitizer	Administration	No. of cases	Included patients	post-BCG (%)	High risk (%)	Light energy (J/cm^2^)
Apolikhin (14)	2007	Russia	Photosens	Intravenous	14	T1G2	NA	14/14	12
Bader (15)	2013	Germany	HAL	Intravesical	17	Intermediate- and high-risk recurrent NMIBC	12/17	15/17	25-100, most 100
Berger (17)	2003	Austria	5-ALA	Intravesical	31	Recurrent NMIBC	10/31	25/31	30-50
Filonenko (20)	2016	Russia	5-ALA	Intravesical	45	NMIBC	NA	41/45	20
Kulkarni (23)	2022	Canada	TLD-1433	Intravesical	6	BCG-unresponsive NMIBC	6/6	6/6	90
Lee (24)	2013	Korea	Radachlorin	Intravenous	34	High grade, BCG refractory or intolerant NMIBC	34/34	34/34	15
Lee (25)	2010	Singapore	Ce6PVP	Intravenous, Intravesical	5	BCG-refractory NMIBC	5/5	5/5	10-24
Nseyo (29)	1998	USA	Photofrin	Intravenous	19	Resistant NMIBC	Most	7/19	10-60
Skyrme (32)	2005	UK	5-ALA	Intravesical	21	Recurrent NMIBC	4/21	19/21	10-25
Sokolov (33)	2005	Russia	Photogeme	Intravesical	86	NMIBC	NA	32/86	10-15
Szygula (34)	2004	Poland	5-ALA	Intravesical	14	T1 NMIBC	14/14	14/14	10
Waidelich (39)	2001	Germany	5-ALA	Oral	24	BCG-Refractory NMIBC	24/24	15/24	60

Ce6PVP, chlorin e6-polyvinylpyrrolidone; HAL, hexaminolevulinate; 5-ALA, 5-aminolevulinic acid; NMIBC, Non-muscle-invasive bladder cancer.

In most studies, PDT was performed once. In Nseyo’s report in 2003, patients were treated by two sequential PDT at baseline and 6 months (28), while in Bader’s study, patients were treated by three sequential PDT within 3 months (15).

### Efficacy of therapeutic PDT

**Figure 3:**
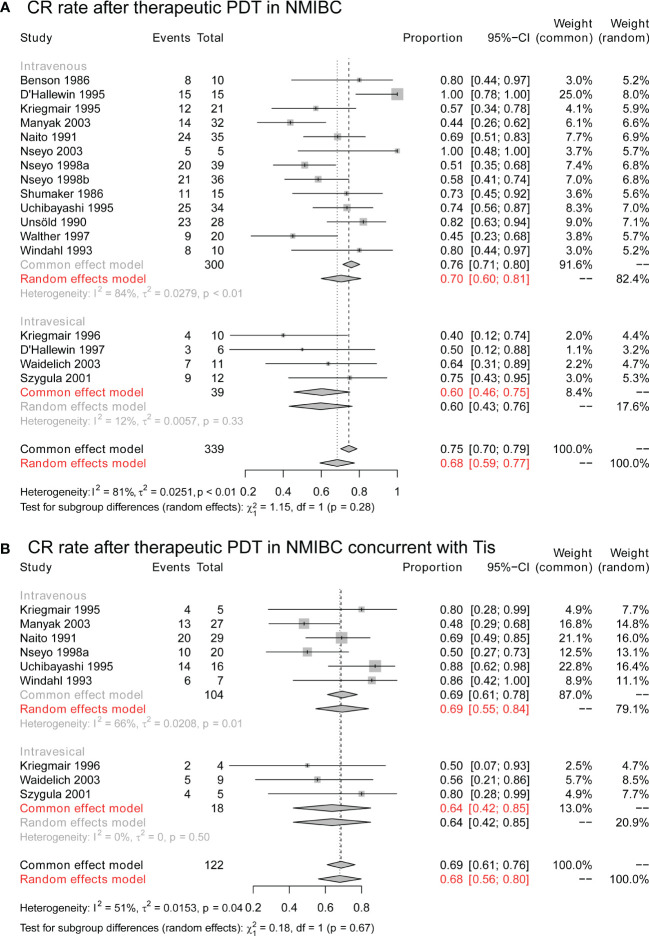
The CR rate of therapeutic PDT in NMIBC. **(A)** The overall CR rate of therapeutic PDT in NMIBC. **(B)** The overall CR rate of therapeutic PDT in NMIBC concurrent with Tis. CR, complete response; PDT, photodynamic therapy; NMIBC, non-muscle-invasive bladder cancer; Tis, tumor in situ.

The CR rates of therapeutic PDT in NMIBC were synthesized to conclude the overall efficacy. As seen in **Figure 3A**, therapeutic PDT achieved a CR rate of 68% (95% CI: 59%-77%), and the CR rates did not differ in patients who received intravenous application of 1^st^ generation photosensitizer and intravesical application of 2^nd^ generation photosensitizer (P = 0.28). Especially, for those who achieved CR, 71% (95% CI: 56%-85%) of them maintained tumor-free at 12 months after PDT (**Figure S1A**), and 38% (95% CI: 12%-64%) of them were tumor-free at 24 months (**Figure S1B**).

Furthermore, as **Figure 3B**, for cases with concurrent Tis, therapeutic PDT still achieved a CR rate of 68% (95% CI: 56%-80%), and CR rates were still similar in intravenous and intravesical groups (P = 0.68). Longterm results suggested a 12-month RFS in 84% (95% CI: 48%-100%) of CR cases (**Figure S2A**), and 13% (95% CI: 1%-32%) of them were tumor-free at 24 months (**Figure S2B**).

### Efficacy of adjuvant PDT

**Figure 4:**
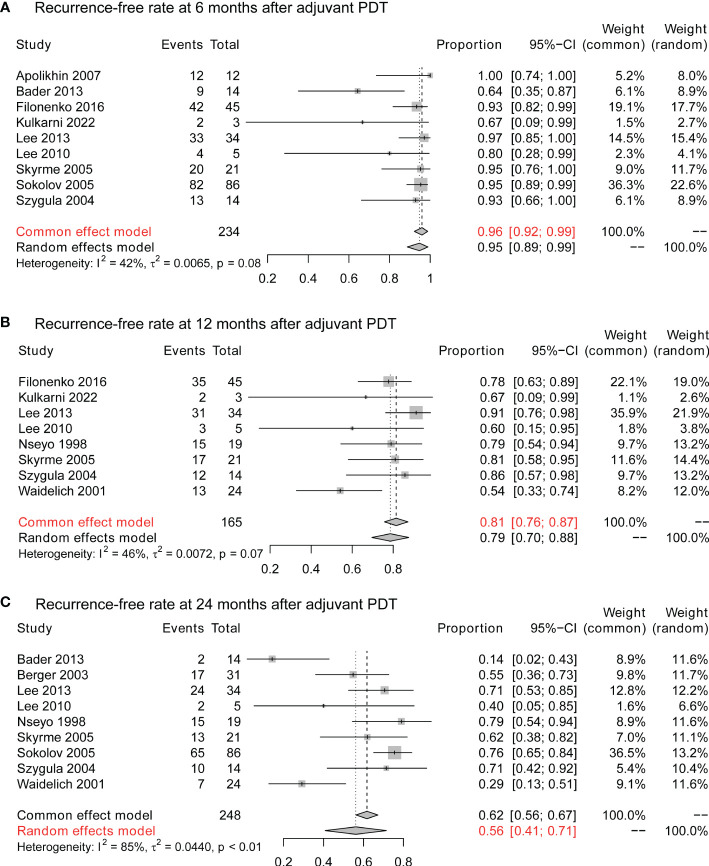
The cancer control of adjuvant PDT in NMIBC. **(A)** The 6-month recurrence-free rate of adjuvant therapy in NMIBC. **(B)** The 12-month recurrence-free rate of adjuvant therapy in NMIBC. **(C)** The 24-month recurrence-free rate of adjuvant therapy in NMIBC. PDT, photodynamic therapy; NMIBC, non-muscle-invasive bladder cancer.

After resection of visible lesions, adjuvant PDT would be applied to reduce the risk of recurrence. At 6 months after adjuvant PDT, 96% (95% CI:92%-99%) of the patients stay tumor-free (**Figure 4A**), and the 1-year tumor-free rate is 81% (95% CI:76%-87%; **Figure 4B**). 2 years after PDT, 56% (95% CI:41%-71%) of patients still present no evidence of tumor recurrence (**Figure 4C**).

For patients who failed intravesical BCG therapy, PDT still provides cancer control for part of patients. At 6 months after adjuvant PDT, 96% (95% CI:91%-100%) did not identify recurrence (**Figure S3A**). At 12 months, 68% (95% CI:51%-86%) of BCG-unresponsive patients remain tumor-free (**Figure S3B**), and the tumor-free rate is 56% (95% CI:32%-81%) at 24 months (**Figure S3C**).

### Complications of PDT

There were several complications secondary to PDT. The local complications, including lower urinary tract symptoms (LUTS) and hematuria, could happen in 91% (95% CI:80%-99%) of patients who underwent PDT, regardless of administration method and photosensitizer type (**Figure S4**).

Skin photosensitivity is reported in 11% (95% CI:5%-20%) of PDT when 1^st^ generation photosensitizers were intravenously used. However, when novel photosensitizers and intravesical PDT were introduced, photosensitivity could be mostly avoided (**Figure S5**). Similarly, bladder contractures were noted in 5% (95% CI:1%-11%) of patients, but were not further reported when novel photosensitizers were intravesically administrated (**Figure S6**).

## Discussion

NMIBC was known for its high recurrence rate. Many efforts were made to reduce the relapse, including surgery reformation (42, 43) and adjuvant treatments (44). But still, part of patients would experience recurrence and progression, and the prognosis of these patients is not satisfying.

PDT is an emerging method to eliminate tumor residues and has been a promising option for BCa. As a non-invasive intervention, PDT is well-tolerated and could be easily and widely used. More importantly, its mechanism is unique. Initially, the effect of PDT was attributed to the release of cytotoxic mediators such as singlet oxygen, which directly trigger anti-tumor effects. As mechanistic knowledge has grown, the multifaceted nature of PDT was understood, which is comprised of anti-vascular actions, multiple cell death pathways, together with innate and adaptive immune stimulations (45).

PDT has been clinically used in the treatment of NMIBC since the 1980s. However, different strategies were implemented by different medical centers, including the indications and treatment protocol. Such as in our analysis, the inclusion criteria were quite different, and the light energy of PDT ranged from 10-100 J/cm^2^. The lack of a standard protocol resulted in the absence of high-quality clinical evidence, which prevents the widespread use of PDT. To the best of our knowledge, no systematic review or meta-analysis is available to provide a comprehensive summary of the clinical utility of PDT in NMIBC.

In this systematic review and meta-analysis, we introduced a pioneering classification of PDT according to the tumor status prior to PDT. Therapeutic PDT is defined as PDT applied to eliminate unresectable lesions. Adjuvant PDT was defined as PDT conducted for prophylaxis of recurrence, before which the visible lesions were eliminated. Notably, PDT was mostly applied to these high-risk patients who failed or were unsuitable for standard treatments.

As shown in **Table 1**, some patients could have multiple or diffuse tumors, and complete resection by conventional TURBT might be unfeasible. For these patients, therapeutic PDT presents a satisfying efficacy rate. As seen in **Figure 3A**, 68% (95% CI: 59%-77%) of these patients were tumor-free after PDT. More surprisingly, the tumor-free status could be long-term in many cases (**Supplement Figure 1**). 12 months after PDT, over half of the CR patients remain tumor-free. 24 months after PDT, about a third of these patients still did not experience recurrence. These results indicate that, when complete resection of tumors is surgically unfeasible, PDT might provide an extra tumor-free survival and delay the RC for part of patients.

Especially, our analysis suggested that Tis patients could also benefit from PDT. As shown in **Figure 3B** and **Supplement Figure 2**, PDT could achieve a CR rate of 68%, and less than a third of these patients could maintain tumor-free for over 2 years. Currently, the recommended treatment for Tis is RC or TURBT combined with intravesical BCG. Intravesical BCG could induce a CR rate of about 75% in Tis patients, and the five-year recurrence-free rate of complete responders was > 50% (46, 47). Statistically, intravesical BCG outperformed PDT in the treatment of Tis and should be preferred for these patients. But for BCG-unresponsive or intolerant patients, PDT could still be an alternative.

PDT could also be an efficient adjuvant option after standard TURBT. In clinical practice, intravesical BCG or chemotherapy is the standard treatment for intermediate- and high-risk NMIBC, especially BCG therapy. For BCG-unresponsive NMIBC, RC is recommended by guidelines (3, 4). Our results indicate that PDT might be an effective treatment for these patients, since PDT could achieve a recurrence-free rate of 68% (95% CI:51%-86%) at 12 months and 56% (95% CI:32%-81%) at 24 months in BCG-unresponsive patients (**Figure S3**). These results have illustrated that PDT is feasible for these refractory NMIBCs which are unsuitable for conventional treatment.

Complications of PDT could be well-managed currently. Local complications including LUTS and hematuria would present in > 90% of patients, but they were mostly transient and could be solved by symptomatic treatments. Skin photosensitivity is common before, but when novel photosensitizers were intravesically administrated, protection against exposure to light was no longer needed (19, 21). Besides, when 5-ALA were orally given, protection from sunlight for 24 hours is enough to avoid phototoxic skin reaction (39), which is much shorter than 4-6 weeks of protection after intravenous administration of the first-generation photosensitizers. Bladder contracture is the most serious complication of PDT due to fibrosis triggered by unspecific light reactions in the normal muscle layer (48). The excessive light dose could be an important reason for it (29). Due to the improvement of photosensitizer selectivity and application of intravesical administration, the bladder contracture was not reported anymore.

The present systematic review and meta-analysis were performed based on previously published literature. Several limitations should be noted. All these studies were single-arm case series. The relatively low quality compromised the strength of our conclusions and the results should be interpreted with caution. Additionally, the inconsistent inclusion criteria in different studies prevent a clear description of the beneficiary. Besides, the heterogeneity in study design, photosensitizer type, and photosensitizer administration did not conclude a present standard protocol of PDT, which is important for the conducting of further high-quality research.

Our systematic review and meta-analysis suggested that PDT could provide certain cancer control for these recurrent, high-risk, or BCG-unresponsive BCa. However, the research on PDT is still inadequate, and the application of PDT in BCa needs much more exploration.

First, further studies with proper design and higher quality are needed to ascertain and extend the beneficiary of PDT. As discussed above, a standard protocol for PDT is urgently needed.

Second, the beneficiary of PDT needs to be determined by future studies. It should be noted that PDT was currently used as a second-line option in high-risk NMIBC. Whether NMIBC, including intermediate- and low-risk, could benefit from the early combination of PDT and intravesical chemotherapy or BCG therapy as first-line treatment needs to be determined.

Third, Last, PDT has unique mechanisms, that might further enhance the efficacy of traditional therapy (49). Adding PDT to conventional intravesical chemotherapy or BCG therapy has been explored for refractory NMIBC (32, 34, 50). Immune checkpoint inhibitors (ICIs) were also emerging options for NMIBC (51, 52). A combination of PDT and ICIs could also be a new way to control NMIBC (53, 54). Further high-quality clinical trials are warranted to confirm the efficacy of these combinations.

Last, Intuitively, enhancing the selectivity and permeability of photosensitizers could be a feasible strategy. Presently, third-generation photosensitizers have been developed by conjugating photosensitizers to molecules that target tumor biomarkers (55). In BCa, cellular and animal experiments of third-generation photosensitizers have been conducted and indicate reinforced PDT efficacy (56, 57).

## Conclusion

Presently, PDT was clinically applied to high-risk NMIBC, including diffuse Tis and those that are resistant to standard of care. Although high-level evidence is still lacking, current studies suggested that both therapeutic and adjuvant PDT present satisfying safety and efficacy. As a promising option for NMIBC, PDT deserves further exploration by future high-quality research.

## Data availability statement

The original contributions presented in the study are included in the article/**Supplementary Material**. Further inquiries can be directed to the corresponding author.

## Author contributions

HL: Data curation, Formal Analysis, Methodology, Software, Validation, Visualization, Writing – original draft. GL: Conceptualization, Data curation, Formal Analysis, Methodology, Software, Visualization, Writing – original draft. JT: Conceptualization, Funding acquisition, Project administration, Supervision, Validation, Writing – review & editing.
