# Supplementary material for: Efficacy and safety of photodynamic therapy for non–muscle-invasive bladder cancer: a systematic review and meta-analysis
Source: Front Oncol. 2023 Oct 4;13:1255632. doi: 10.3389/fonc.2023.1255632 (PMC10584312; doi:10.3389/fonc.2023.1255632)

[Supplement Figure 1: Longterm recurrence-free rate of therapeutic PDT in NMIBC. 2](#_Toc142135037)

[Supplement Figure 2: Longterm recurrence-free rate of therapeutic PDT in NMIBC concurrent with Tis. 3](#_Toc142135038)

[Supplement Figure 3: The cancer control of adjuvant PDT in BCG-unresponsive NMIBC. 4](#_Toc142135039)

[Supplement Figure 4: Incidence of local complications after PDT in NMIBC. 5](#_Toc142135040)

[Supplement Figure 5: Incidence of skin photosensitivity after PDT in NMIBC. 6](#_Toc142135041)

[Supplement Figure 6: Incidence of bladder contracture after PDT in NMIBC. 7](#_Toc142135042)

# Supplement Figure 1: Longterm recurrence-free rate of therapeutic PDT in NMIBC.

(A) The 12-month recurrence-free rate of therapeutic PDT in NMIBC. (B) The 24-month recurrence-free rate of therapeutic PDT in NMIBC. PDT: photodynamic therapy; NMIBC: non-muscle-invasive bladder cancer.


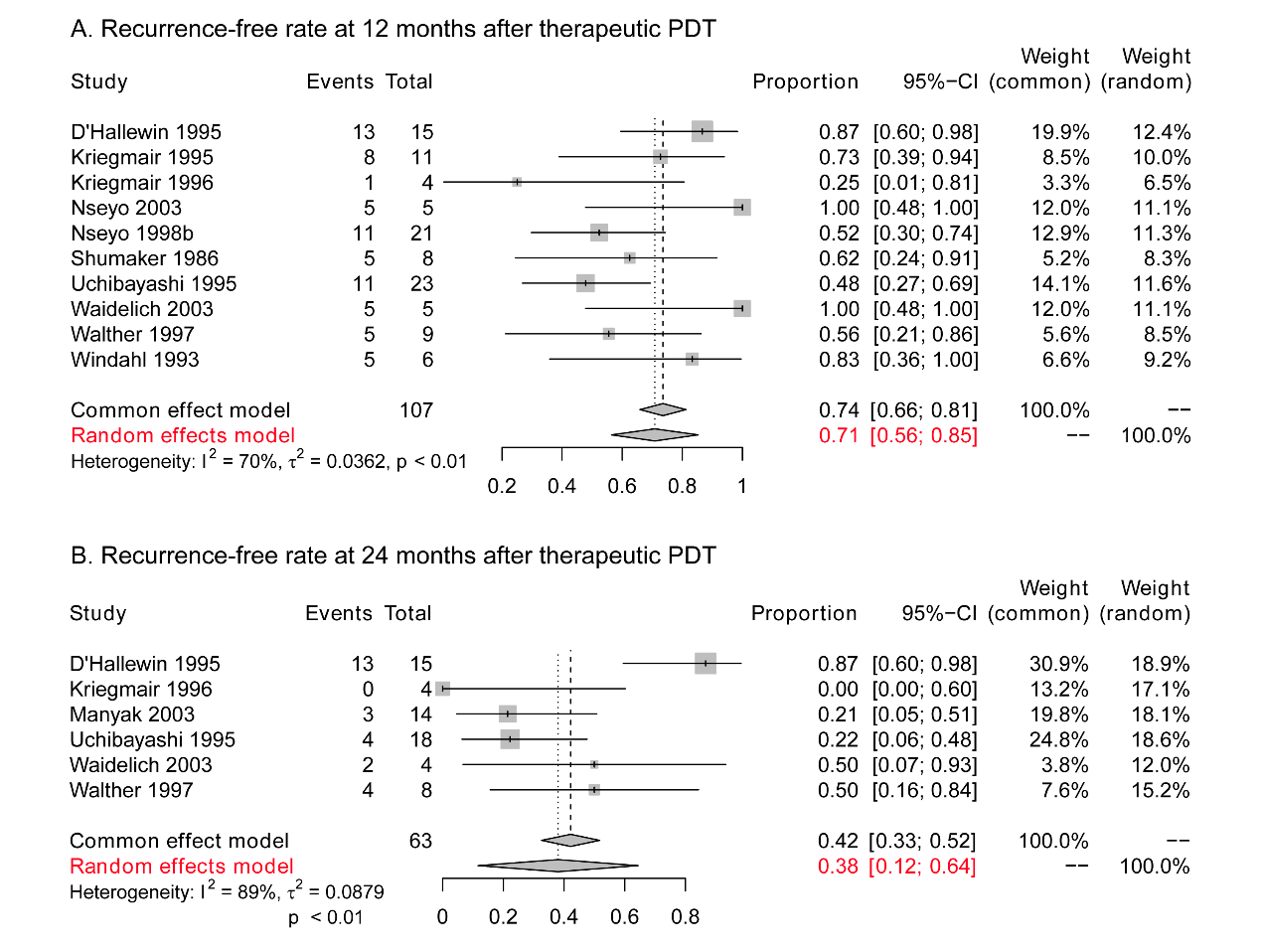


# Supplement Figure 2: Longterm recurrence-free rate of therapeutic PDT in NMIBC concurrent with Tis.

(A) The 12-month recurrence-free rate of therapeutic PDT in NMIBC concurrent with Tis. (B) The 24-month recurrence-free rate of therapeutic PDT in NMIBC concurrent with Tis. PDT: photodynamic therapy; NMIBC: non-muscle-invasive bladder cancer; Tis: tumor in situ.


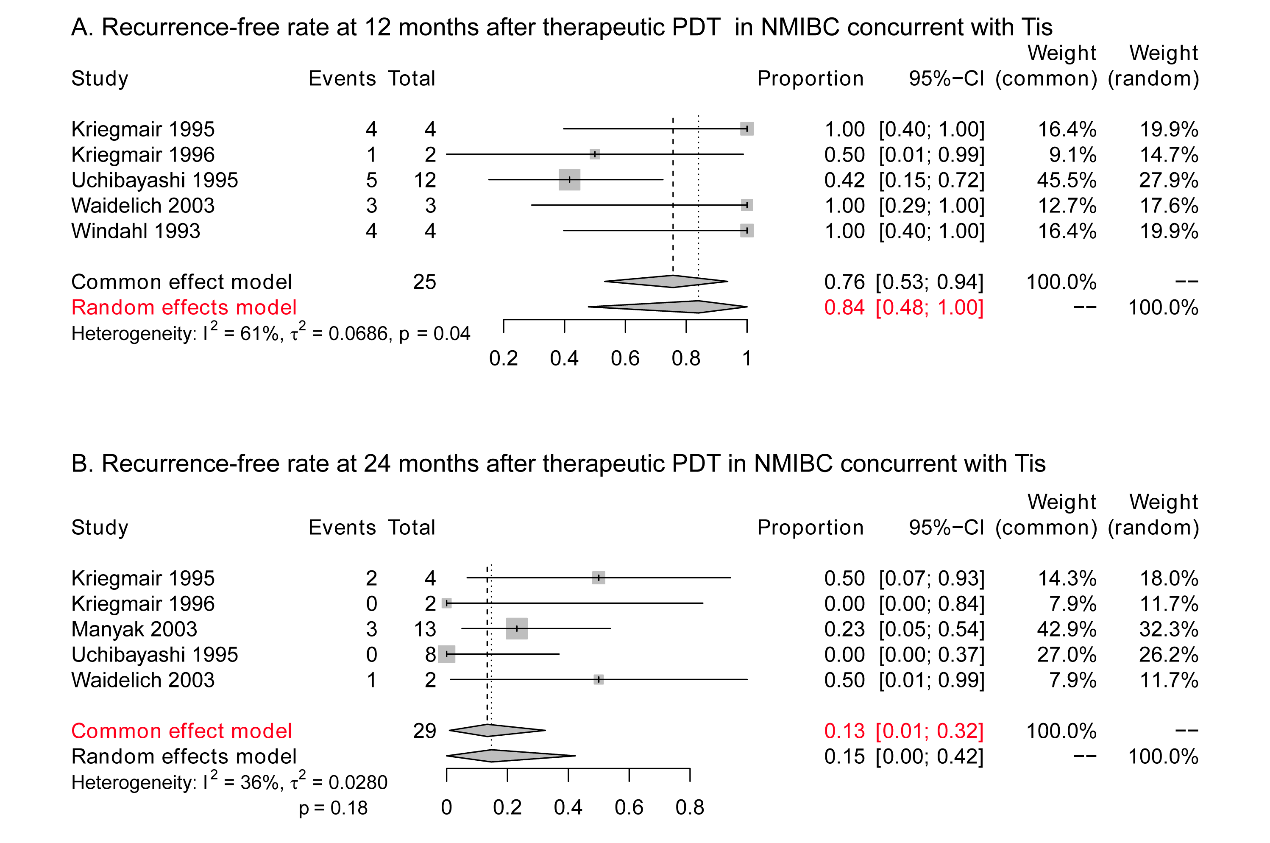


# Supplement Figure 3: The cancer control of adjuvant PDT in BCG-unresponsive NMIBC.

(A) The 6-month recurrence-free rate of adjuvant therapy in BCG-unresponsive NMIBC. (B) The 12-month recurrence-free rate of adjuvant therapy in BCG-unresponsive NMIBC. (C) The 24-month recurrence-free rate of adjuvant therapy in BCG-unresponsive NMIBC. PDT: photodynamic therapy; NMIBC: non-muscle-invasive bladder cancer.


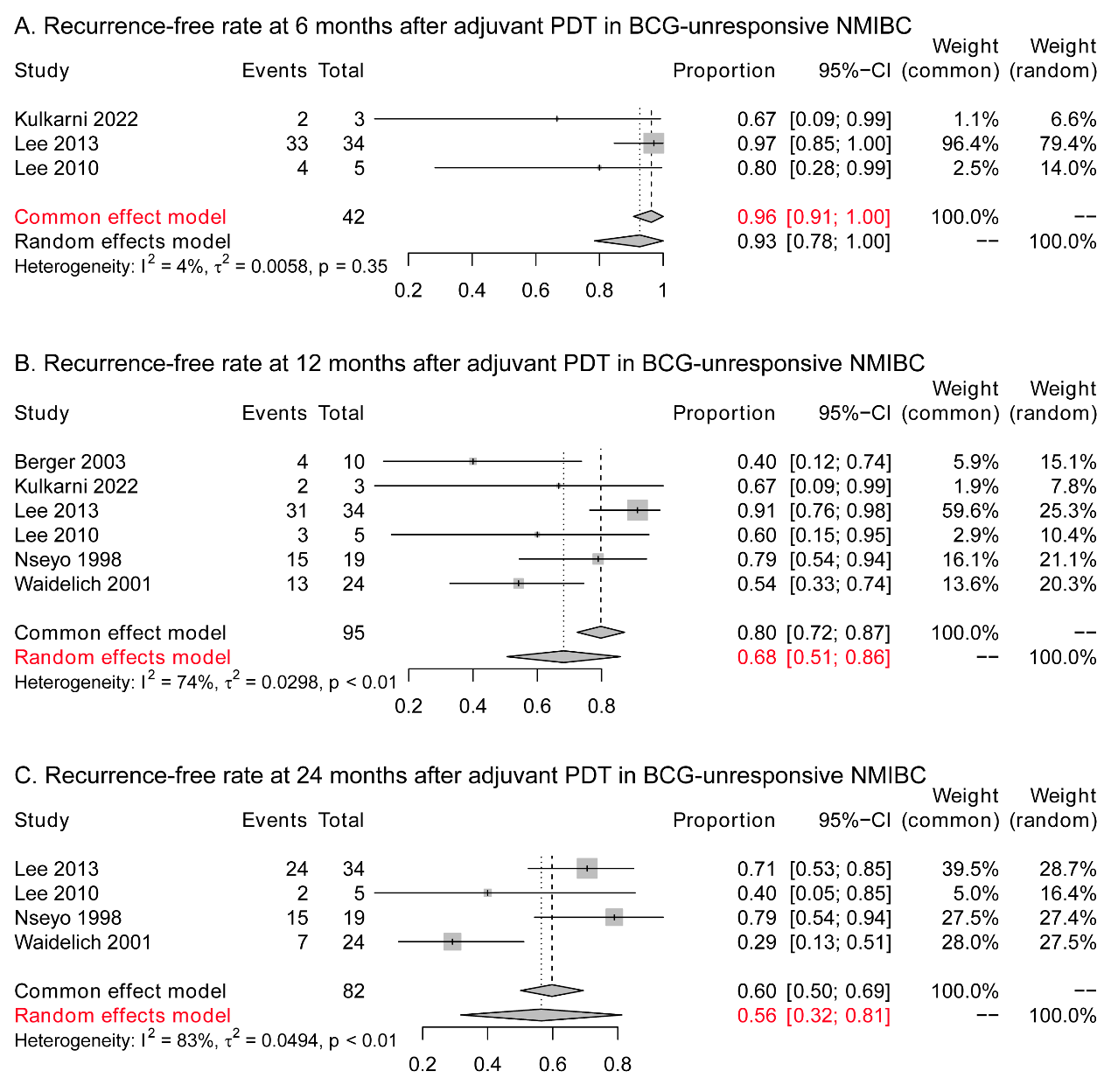


# Supplement Figure 4: Incidence of local complications after PDT in NMIBC.

(A) Incidence of local complications stratified by photosensitizers. (B) Incidence of local complications stratified by drug administration.


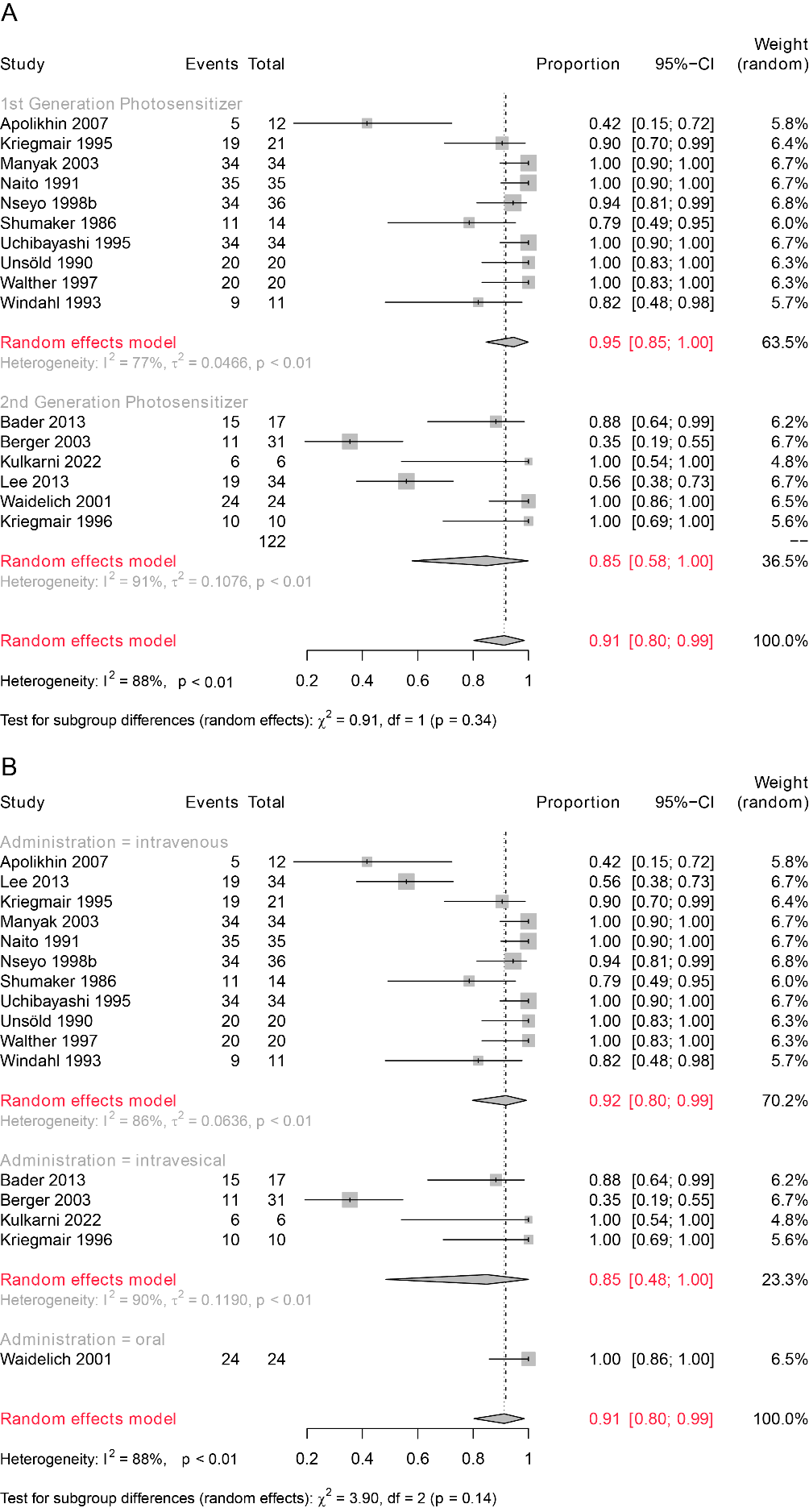


# Supplement Figure 5: Incidence of skin photosensitivity after PDT in NMIBC.


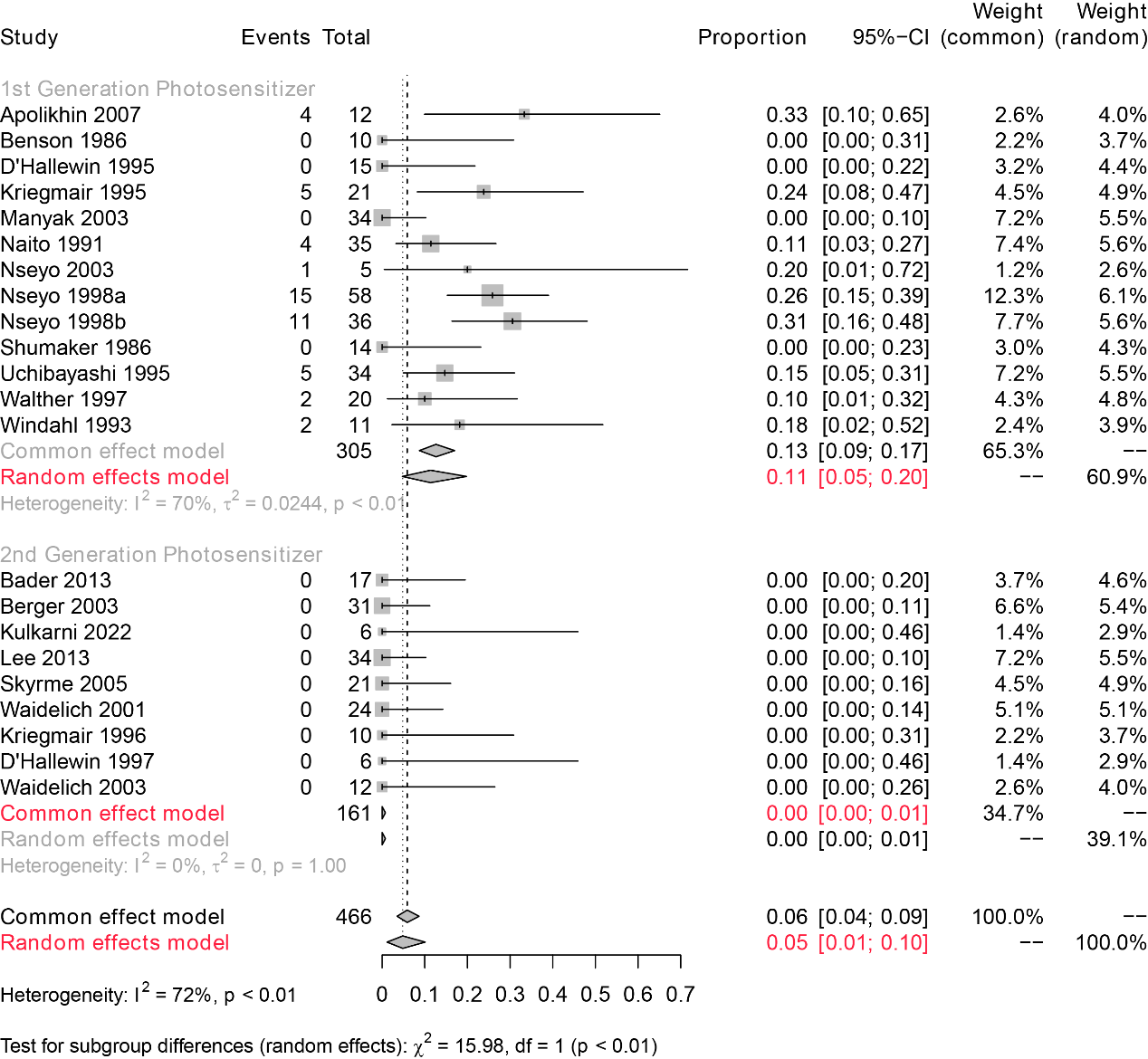


# Supplement Figure 6: Incidence of bladder contracture after PDT in NMIBC.


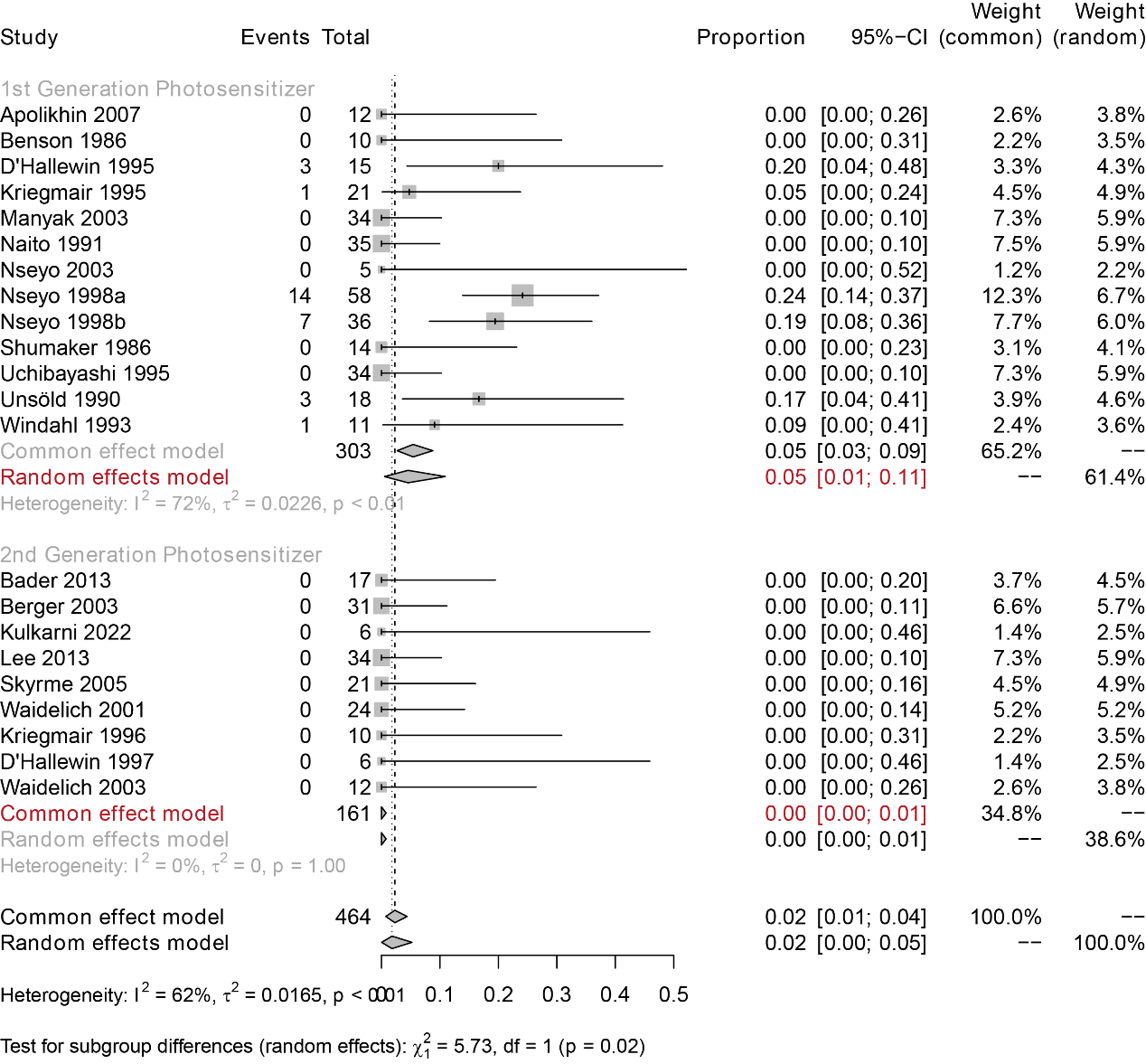

Supplement: Supplementary file 1 [file DataSheet_1.docx]
